# Characterization of Premigration and Postmigration Multidomain Factors and Psychosocial Health Among Refugee Children and Adolescents After Resettlement in Australia

**DOI:** 10.1001/jamanetworkopen.2023.5841

**Published:** 2023-04-06

**Authors:** Lan Guo, Li Li, Ke Xu, Wanxin Wang, Yanyan Ni, Wenyan Li, Jianhua Gong, Ciyong Lu, Wei-Hong Zhang

**Affiliations:** 1 Department of Medical Statistics and Epidemiology, School of Public Health, Sun Yat-sen University, Guangzhou, People’s Republic of China; 2 Luohu District Maternity and Children Health Care Hospital, Shenzhen, People’s Republic of China; 3 School of Public Health, The University of Hong Kong, Special Administrative Region, People’s Republic of China; 4 Faculty of Medicine and Health Sciences, Department of Public Health and Primary Care, Ghent University, Ghent, Belgium

## Abstract

**Importance:**

A better understanding of the psychosocial health of resettled child and adolescent refugees and associated premigration and postmigration factors may help this population integrate effectively.

**Objective:**

To estimate the associations of premigration and postmigration multidomain factors with psychosocial health after resettlement among young refugees of different ages.

**Design, Setting, and Participants:**

This cross-sectional study used wave 3 data from the Building a New Life in Australia (BNLA) cohort study, as they represented the first time a BNLA study included a child module targeting children and adolescents in the migrating unit as a nested component of the broader study. The study population consisted of children aged 5 to 10 years and adolescents aged 11 to 17 years. The caregivers of the children, the adolescents themselves, and the adolescents’ caregivers were invited to complete the child module. Wave 3 data were collected from October 1, 2015, to February 29, 2016. Statistical analysis was performed from May 10 to September 21, 2022.

**Exposures:**

Premigration and postmigration multidomain factors, including individual (child and caregiver), family, school, and community levels, were measured.

**Main Outcomes and Measures:**

Social and emotional adjustment and posttraumatic stress disorder (PTSD) were the dependent variables measured by the Strengths and Difficulties Questionnaire (SDQ) and an 8-item PTSD scale. Weighted multilevel linear or logistic regression models were used.

**Results:**

Of the 220 children aged 5 to 10 years (mean [SD] age, 7.4 [2.0] years), 117 (53.2%) were boys; of the 412 adolescents aged 11 to 17 years (mean [SD] age, 14.1 [2.0] years), 215 (52.2%) were boys. Among the children, compared with no exposure, exposure to premigration traumatic events (β = 2.68 [95% CI, 0.51-4.85]) and having family conflicts after resettlement (β = 6.30 [95% CI, 2.97-9.64]) were positively associated with SDQ total difficulties score; school achievement was negatively associated with SDQ total difficulties score (β = −5.02 [95% CI, −9.17 to −0.87]). Among the adolescents, being treated unfairly (β = 3.32 [95% CI, 1.41-5.22]) and parenting harshness after resettlement (β = 0.25 [95% CI, 0.11-0.40]) were positively associated with SDQ total difficulties score; engagement in extracurricular activities (β = −3.67 [95% CI, −6.83 to −0.50]) was negatively associated with SDQ total difficulties score. Exposure to premigration traumatic events (adjusted odds ratio [aOR], 2.49 [95% CI, 1.10-5.63]), being treated unfairly (aOR, 3.77 [95% CI, 1.60-8.91]), and facing English language barriers (aOR, 6.41 [95% CI, 1.98-20.79]) after resettlement were positively associated with the presence of PTSD.

**Conclusions and Relevance:**

In this study of refugee children and adolescents, apart from premigration traumatic experiences, several postmigration family- and school-related factors and social integration factors were associated with psychosocial health after resettlement. The findings suggest that family- and school-centered psychosocial care and social integration programs targeting related stressors merit increased attention for improving the psychosocial health of refugee children and adolescents after resettlement.

## Key Points

**Question:**

What premigration and postmigration multidomain factors are associated with psychosocial health among refugee children and adolescents after resettlement in Australia?

**Findings:**

This cross-sectional study of 220 refugee children and 412 refugee adolescents found that although discrepancies in the associations of individual-, family-, school-, and community-level factors with psychosocial health were observed between age groups, premigration traumatic experiences and some postmigration family- and school-related factors and social integration factors were associated with psychosocial health after resettlement.

**Meaning:**

These findings support the potential benefits of promoting family- and school-centered psychosocial care and social integration programs targeting related stressors in improving the psychosocial health of refugee children and adolescents after resettlement.

## Teaser

This cross-sectional study estimates the associations of premigration and postmigration multidomain factors with psychosocial health after resettlement among refugee children and adolescents in the Building a New Life in Australia cohort.

## Introduction

By the end of 2021, an estimated 94.7 million people were forcibly displaced worldwide due to persecution, conflict, human rights violations, violence, or events seriously disrupting the public order.^1^ Of all forcibly displaced people, children and adolescents accounted for 42%.^1^ Today, attention to children and adolescent health is an important issue worldwide.^2^ Previous evidence^3,4,5,6^ has shown that compared with general children and adolescents, refugee children and adolescents often experience significant physical and mental challenges during displacements, endure continuing adversities even after arrival, and hence are more vulnerable to psychosocial health problems. Given the increasing pressure for host countries to resettle a greater number of refugees, it is important to estimate young refugees’ psychosocial health status and associated factors to promote positive resettlement outcomes.

Although exposure to violence or traumatic events has been shown to be a key risk factor for refugees’ psychosocial health,^7^ based on the framework proposed by Zimmerman et al,^8^ many diverse factors (eg, social support or some protective policies) may also have influenced their psychological functioning during the entire migration process. However, although services or intervention strategies exist to better settle refugees, they are still at elevated risk of poor psychosocial health, and fewer services and strategies focus on the needs of refugee children and adolescents.^9^ The resettlement process for children and adolescents can be quite gradual, not only because of the challenges of complex legal immigration processes but also the challenges of sociocultural adaptation to enormous cultural, social, and linguistic differences.^9,10^ Several multidomain factors in dispersive studies, including those related to the individual (child and caregiver),^4,7,11,12,13^ family (eg, family structure or family relationship),^14,15^ school (eg, school adaptation or peer support),^16,17^ and community (eg, social relationships or social integration)^18,19^ contexts, have been reported to be associated with psychosocial health in refugee children and adolescents. The susceptibility to premigration and postmigration stressors and protective systems affecting the resilience of children and adolescents might be slightly different.^20^ However, the results of previous studies have not been conclusive, and there is a scarcity of studies that comprehensively estimate premigration and postmigration multidomain factors associated with the psychosocial health of resettled refugee children and adolescents. In addition, postmigration stressors from caregivers or families (eg, financial status or social integration stressors) are relatively less considered. Therefore, using data from the Building a New Life in Australia (BNLA) cohort study, one of the first and largest global cohort studies of refugees and their families,^21^ this study aimed to estimate the associations of premigration and postmigration multidomain factors with psychosocial health after resettlement among refugee children and adolescents.

## Methods

### Study Design and Data

The BNLA study is a longitudinal national refugee-based cohort study that traced the settlement journey of recently resettled humanitarian migrants in Australia over 5 waves (2013-2018) and investigated outcomes and risk factors related to this process.^22,23^ Participants in the BNLA study were recruited from 11 Australian sites covering major cities and regional areas, with the highest number of humanitarian migrants settling between November 1, 2010, and October 31, 2011. Details about the BNLA study sampling and follow-up procedures have been published elsewhere.^22^ All participants in the BNLA study provided voluntary written consent. Ethical approval was obtained from the Australian Institute of Family Studies Human Research Ethics Committee. This cross-sectional study followed the Strengthening the Reporting of Observational Studies in Epidemiology (STROBE) reporting guideline.

In the present study, we used wave 3 data from the BNLA project, which were collected from October 1, 2015, to February 29, 2016, as they represented the first time a BNLA study included a child module targeting children and adolescents aged 5 to 17 years in the migrating unit as a nested component of the broader study.^21,23^ In wave 3, initial sampling for the child module occurred by randomly selecting up to 2 children aged 5 to 17 years in each household. In households with multiple children but only 1 child aged 11 to 17 years, the eldest child was recruited, and 1 younger child aged 5 to 10 years was randomly selected. In households with only younger children, 2 children aged 5 to 10 years were randomly selected. The caregivers of children aged 5 to 10 years were invited to complete the child module, which was administered via pencil and paper. Adolescents aged 11 to 17 years and their caregivers were invited to complete the child module. The Figure describes the recruitment process of the participants (principal and secondary applicants) in wave 1 and the subsequent recruitment of caregivers, children, and adolescents in wave 3. The detailed process of participant selection is listed in eMethods 1 in Supplement 1.

**Figure.**
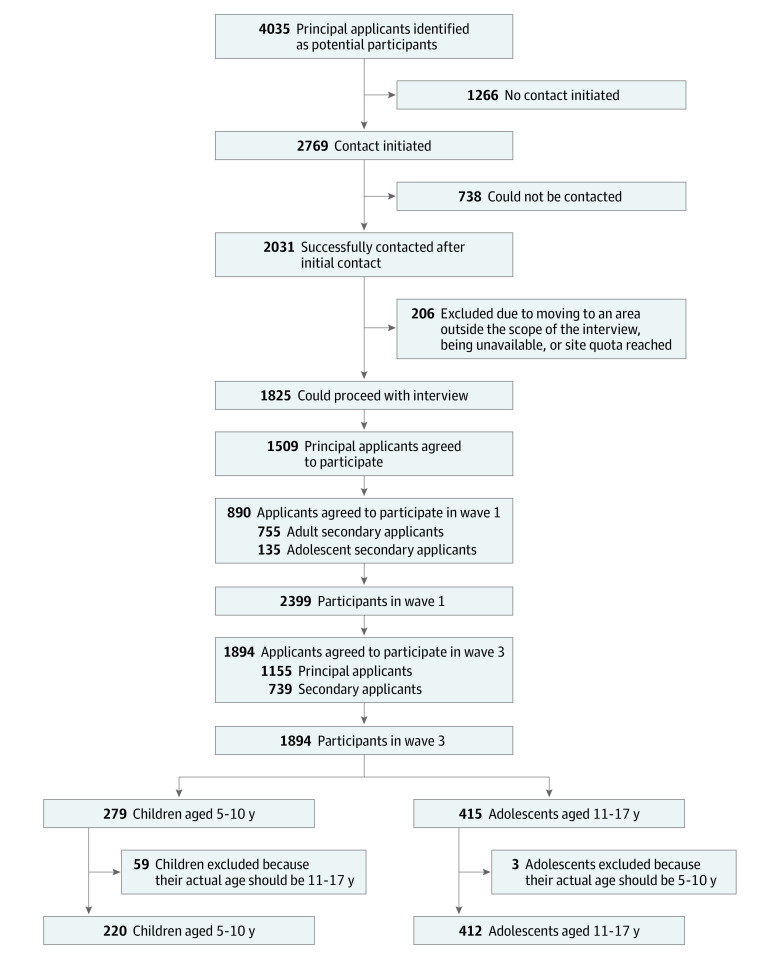
Study Flowchart Recruitment process of participants (principal and secondary applicants) in wave 1 (October 1, 2013, to February 28, 2014) and the subsequent recruitment of caregivers, children, and adolescents in wave 3 (October 1, 2015, to February 29, 2016).

### Measures

#### Independent Variables

Details on the definition and code of these independent variables are listed in eMethods 2 in Supplement 1. Individual domain factors included child-level factors (ie, age, sex, and premigration stressors, including children’s experience of exposure to traumatic events and experience of safety- or life-threatening events), postmigration factors (including being treated unfairly, having English language barriers, physical health, and physical activity), and caregiver-level factors (ie, geographic region of caregiver’s birth and caregiver’s postmigration stressors).

Family domain factors consisted of family structure and parenting style after resettlement. School domain factors incorporated school awards, school achievement, and school absenteeism after resettlement. Community domain factors included engagement in extracurricular activities, ethnic or religious community support, neighborhood friendliness, and perceptions of neighborhood safety after resettlement. This study summarized different domains regarding the aforementioned variables in the eFigure in Supplement 1.

#### Dependent Variables

Our study used emotional and behavioral problems and posttraumatic stress disorder (PTSD) as the dependent variables, and details about the measurements are listed in eMethods 2 in Supplement 1. Emotional and behavioral problems were assessed by the parent-report and self-report versions of the Strengths and Difficulties Questionnaire (SDQ), which have been validated and widely used with satisfactory psychometric properties.^24,25,26,27^ The SDQ consists of 25 items operationalizing 5 subscales: prosocial behavior, peer problems, emotional problems, conduct problems, and hyperactivity. Each subscale’s score ranged from 0 to 10. The total difficulties score indicates the sum of all subscales excluding prosocial behavior,^28^ with higher scores representing a greater level of difficulties. In contrast, lower scores on the prosocial behavior subscale indicated greater psychological problems. The related data from the SDQ parent-reported version for children aged 5 to 10 years and the SDQ self-reported version for adolescents aged 11 to 17 years were used in this study.

We assessed PTSD using an 8-item PTSD scale (PTSD-8) derived from the Harvard Trauma Questionnaire Part IV, which has been validated and used cross-culturally.^29^ The PTSD-8 covers all 3 symptom clusters of the *Diagnostic and Statistical Manual of Mental Disorders (Fourth Edition)* (*DSM-IV*)–based PTSD diagnosis (ie, intrusion, avoidance, and hyperarousal). The PTSD-8 consists of 8 items, each addressing the frequency of specific symptoms of PTSD over the past week. Responses for each item are rated using a 4-point Likert scale (1 indicates not at all; 2, rarely; 3, sometimes; and 4, most of the time). The total score varied from 8 to 32. Posttraumatic stress disorder was determined to be present if each PTSD symptom cluster had at least 1 item score of 3 (sometimes) or higher.^29^

### Statistical Analysis

Statistical analysis was performed from May 10 to September 21, 2022. First, we compared the information reported by caregivers and adolescents aged 11 to 17 years (Table 1). If responses from both caregiver and adolescent reports were available, then self-reported data from the adolescents were used. If data were available from adolescent self-reports only or caregiver reports only, the available information was analyzed.

**Table 1.** Sample Characteristics in Individual-, Family-, School-, and Community-Level Domains Among Refugee Children and Adolescents in the Building a New Life in Australia Project

Characteristic	Age category, No. (%)^a^	
	Children, 5-10 y (n = 220)	Adolescents, 11-17 y (n = 412)
**Individual domain**		
Child level		
Age, mean (SD), y^b^	7.4 (2.0)	14.1 (2.0)
Sex^b^		
Boys	117 (53.2)	215 (52.2)
Girls	103 (46.8)	197 (47.8)
Premigration stressors^c^		
Exposed to traumatic events	37 (16.8)	83 (20.1)
Safety or life badly threatened	31 (14.1)	69 (16.7)
Treated unfairly^c^	NR	92 (22.3)
English language barriers^b^	15 (6.8)	38 (9.2)
Rating of physical health, mean (SD)^b^^,^^d^	4.1 (0.9)	4.1 (0.9)
Physical activity in past week, median (IQR), d^b^	1.0 (0-3.0)	1.0 (0-3.0)
Caregiver level		
Geographic region of caregiver’s birth^e^		
Middle East	123 (58.0)	186 (53.6)
Central Asia (only Afghanistan)	48 (22.6)	115 (33.1)
Southern Asia	20 (9.4)	20 (5.8)
Southeast Asia (only Myanmar)	9 (4.2)	11 (3.2)
Sub-Saharan Africa	5 (2.4)	11 (3.2)
North Africa	7 (3.3)	4 (1.2)
Caregiver’s postmigration stressors^e^		
No. of economic stressors		
0	72 (34.0)	125 (37.3)
1	63 (29.7)	69 (20.6)
2	47 (22.2)	81 (24.2)
3	30 (14.2)	60 (17.9)
No. of concerns about family in Australia		
0	124 (58.5)	206 (61.5)
1	51 (24.1)	85 (25.4)
2	37 (17.5)	44 (13.1)
No. of social integration stressors		
0	71 (33.5)	109 (32.5)
1	110 (51.9)	192 (57.3)
2	31 (14.6)	34 (10.1)
Discrimination	6 (2.8)	16 (4.8)
Loneliness	25 (11.8)	53 (15.8)
Family conflicts in Australia	10 (4.7)	20 (5.8)
Problems with adjustment to life in Australia	31 (14.6)	51 (14.7)
Time between arrival in Australia and interview, y^d^		
1 to <2	8 (3.8)	8 (2.3)
2 to <3	187 (88.2)	329 (94.8)
≥3 to 5	17 (8.0)	10 (2.9)
**Family domain**		
Family structure^e^		
Couple family with children aged <18 y	136 (64.2)	108 (31.1)
Couples with children aged <18 y and other families	47 (22.2)	140 (40.3)
Single with children aged <18 y	19 (9.0)	25 (7.2)
Single with children aged <18 y and other families	8 (3.8)	69 (19.9)
Parenting style^e^		
Parenting warmth score, median (IQR)^f^	21.0 (18.0-24.0)	20.0 (17.0-23.0)
Parenting harshness score, median (IQR)^f^	8.0 (6.0-11.0)	8.0 (6.0-11.0)
**School domain**		
Achievement award last year^c^	NR	278 (67.5)
School achievement average or above average^e^	207 (94.1)	384 (94.3)
School absenteeism per 4 wk, mean (SD), d^e^	0.9 (1.6)	1.3 (2.4)
**Community domain**		
Engagement in extracurricular activities^c^	NR	360 (87.4)
Ethnic or religious community support^e^		
No	86 (40.6)	164 (49.0)
Sometimes	40 (18.9)	68 (20.3)
Yes	86 (40.6)	103 (30.7)
Neighborhood friendly^e^	203 (92.8)	303 (95.9)
Neighborhood feels safe^e^	206 (97.2)	323 (95.8)

Abbreviation: NR, not reported.

^a^ Numbers might not add up to the column total because of missing data. Data are presented as No. (%) for categorical variables. The normality of the distribution of continuous variables was tested by a 1-sample Kolmogorov-Smirnov test. Continuous variables with a normal distribution are presented as the mean (SD); nonnormal variables are reported as the median (IQR).

^b^ Information came from both caregiver and child reports; caregiver-reported information was used for children aged 5 to 10 years and child-reported information for children aged 11 to 17 years.

^c^ Indicates child-reported information.

^d^ Scores range from 1 to 5, with higher scores indicating better health.

^e^ Indicates caregiver-reported information.

^f^ Scores range from 5 to 25, with higher scores indicating more warmth or more harshness.

Descriptive analyses stratified by age groups were used to describe the sample characteristics, and data were presented as number (percentage), mean (SD), or median (IQR) as appropriate. Multilevel regression model analysis in which the migrating unit was the level 1 unit and factors in multiple domains were level 2 units was performed using the GLMMIX procedure in SAS, version 9.2 (SAS Institute Inc), with a random effect on individuals; the wave 3 survey weights were also used in this procedure. Weighted multilevel linear or logistic regression models were performed according to the dependent variable, and unstandardized regression coefficients or odds ratios (ORs) with 95% CIs were reported. In multivariable regression models, variables with *P* < .10 in univariable analyses (eTables 2-4 in Supplement 1) or widely reported in the literature (eg, sex and age) were incorporated to determine the independent associations for psychosocial health. Moreover, to compare the relative importance of different independent variables, we also calculated standardized regression coefficients of multivariable multilevel regression models. The relative importance means the magnitude of an independent variable’s influence on the dependent variable, and the rank order of each independent variable suggests its relative importance from the greatest to the smallest.^30^ All statistical analyses were conducted using SAS, version 9.2. Statistical significance was evaluated at 2-tailed *P* <. 05.

## Results

Table 1 summarizes the information provided by caregivers and adolescents. Of the 220 children aged 5 to 10 years (mean [SD] age, 7.4 [2.0] years), 117 (53.2%) were boys and 103 (46.8%) were girls; 37 (16.8%) reported exposure to premigration traumatic events; and most (204 [96.2%]) had more than 2 years of resettlement. Of the 412 adolescents aged 11 to 17 years (mean [SD] age, 14.1 [2.0] years), 215 (52.2%) were boys and 197 (47.8%) were girls; 83 (20.1%) reported exposure to premigration traumatic events; and most (339 [97.7%]) reported more than 2 years of resettlement.

As shown in eTable 1 in Supplement 1, the mean (SD) total difficulties SDQ scores were 8.6 (6.5) in the children aged 5 to 10 years and 8.8 (5.6) in the adolescents aged 11 to 17 years. Many participants reported having peer problems (50 of 206 [24.3%] in children and 97 of 408 [23.8%] in adolescents). Among the adolescents, 59 (14.3%) met the criteria for PTSD.

The results of weighted univariable multilevel regression models for SDQ total difficulties score, prosocial behavior score, and PTSD status are reported in eTables 2 to 4 in Supplement 1. Table 2 shows that among children aged 5 to 10 years, compared with no exposure, exposure to premigration traumatic events (β = 2.68 [95% CI, 0.51-4.85]) and having family conflicts after resettlement (β = 6.30 [95% CI, 2.97-9.64]) were positively associated with SDQ total difficulties score; school achievement (β = −5.02 [95% CI, −9.17 to −0.87]) was negatively associated with SDQ total difficulties score. Among adolescents aged 11 to 17 years, being treated unfairly (β = 3.32 [95% CI, 1.41-5.22]) and parenting harshness (β = 0.25 [95% CI, 0.11-0.40]) after resettlement were positively associated with SDQ total difficulties score; engagement in extracurricular activities (β = −3.67 [95% CI, −6.83 to −0.50]) was negatively associated with SDQ total difficulties score. Based on the standardized β estimate, among children aged 5 to 10 years, the top 2 positively associated factors for SDQ total difficulties score were exposure to premigration traumatic events and having family conflicts after resettlement; among adolescents aged 11 to 17 years, the top 2 were being treated unfairly and parenting harshness after resettlement.

**Table 2.** Factors Across Multiple Domains Associated With Strengths and Difficulties Questionnaire Total Difficulties Among Refugee Children and Adolescents in the Building a New Life in Australia Project^**a**^

Factor	Strengths and Difficulties Questionnaire total difficulties, age category					
**Children, 5-10 y (n = 220)**			**Adolescents, 11-17 y (n = 412)**		
**Unstandardized β coefficient (95% CI)**	***P* value**	**Standardized β coefficient**	**Unstandardized β coefficient (95% CI)**	***P* value**	**Standardized β coefficient **
**Individual domain**						
Child level						
Age (1-y increase)^b^	0.29 (−0.05 to 0.64)	.09	0.09	−0.06 (−0.39 to 0.28)	.72	−0.02
Sex (reference category is girls)^b^	0.72 (−1.07 to 2.50)	.43	0.06	−1.56 (−2.92 to −0.21)	.02	−0.15^c^
Premigration stressors^d^						
Exposed to traumatic events (reference category is no)	2.68 (0.51 to 4.85)	.02	0.15^c^	1.35 (−0.68 to 3.38)	.19	0.10
Safety or life badly threatened (reference category is no)	NA	NA	NA	1.14 (−0.95 to 3.24)	.28	0.08
Treated unfairly in last 6 mo (reference category is no)^d^	NA	NA	NA	3.32 (1.41 to 5.22)	.001	0.23^c^
Rating of physical health (1-U increase)^b^	−2.68 (−4.07 to −1.29)	<.001	−0.40^c^	−0.94 (−1.83 to −0.05)	.04	−0.15^c^
Physical activity in past week (1-d increase)^b^	0.19 (−0.20 to 0.57)	.34	0.07	0.03 (−0.18 to 0.24)	.81	0.02
Caregiver level						
Geographic region of caregiver’s birth^e^						
North Africa	1 [Reference]	NA	NA	1 [Reference]	NA	NA
Middle East	1.40 (−0.50 to 3.29)	.15	0.11	1.01 (−3.27 to 5.29)	.64	0.09
Southeast Asia (only Myanmar)	−2.74 (−7.32 to 1.85)	.24	−0.09	2.75 (−2.94 to 8.44)	.34	0.11
Southern Asia	1.23 (−1.15 to 3.61)	.31	0.08	1.16 (−4.25 to 6.58)	.67	0.07
Central Asia (only Afghanistan)	2.71 (0.35 to 5.07)	.03	0.16	1.41 (−3.20 to 6.03)	.55	0.12
Sub-Saharan Africa	1.54 (−4.27 to 7.36)	.60	0.02	−1.72 (−6.31 to 2.88)	.46	−0.07
Caregiver’s postmigration stressors^e^						
No. of concerns about family in Australia (1-U increase)	NA	NA	NA	0.49 (−0.45 to 1.43)	.30	0.07
Family conflicts in Australia (reference category is no)	6.30 (2.97 to 9.64)	<.001	0.19^c^	NA	NA	NA
Time between arrival in Australia and interview, y^e^						
1 to <2	NA	NA	NA	1 [Reference]	NA	NA
2 to <3	NA	NA	NA	5.62 (−0.69 to 11.92)	.08	0.22
≥3	NA	NA	NA	4.24 (−1.00 to 9.48)	.11	0.19
**Family domain**						
Parenting harshness (1-U increase)^e^	0.26 (−0.01 to 0.52)	.06	0.16	0.25 (0.11 to 0.40)	.001	0.20^c^
**School domain**						
School achievement average or above average (reference category is no)^e^	−5.02 (−9.17 to −0.87)	.02	−0.18^c^	NA	NA	NA
School absenteeism (1-d increase)^e^	0.31 (−0.13 to 0.74)	.17	0.08	0.19 (−0.03 to 0.41)	.10	0.08
**Community domain**						
Extracurricular engagement (reference category is no)^d^	NA	NA	NA	−3.67 (−6.83 to −0.50)	.02	−0.21^c^
Neighborhood friendly (reference category is disagree)^e^	−3.21 (−7.93 to 1.50)	.18	−0.09	NA	NA	NA
Neighborhood feels safe (reference category is disagree)^e^	0.59 (−2.29 to 3.47)	.69	0.02	−2.36 (−5.12 to 0.40)	.09	−0.09

Abbreviation: NA, not available or applicable.

^a^ Weighted multivariable multilevel regression models were performed in which the migrating unit was the level 1 unit and factors in multiple domains were level 2 units.

^b^ Information came from both caregiver and child reports; caregiver-reported information was used for children aged 5 to 10 years, and adolescent-reported information for adolescents aged 11 to 17 years.

^c^ Significant standardized regression coefficients were ranked from the first to the fourth or fifth in each model.

^d^ Indicates child-reported information.

^e^ Indicates caregiver-reported information.

Table 3 demonstrates that facing English language barriers (β = −3.15 [95% CI, −6.10 to −0.20]) after resettlement was negatively associated with SDQ prosocial behavior score among children aged 5 to 10 years. Among adolescents aged 11 to 17 years, the number of social integration stressors (β = −0.40 [95% CI, −0.74 to −0.06]) after resettlement was negatively associated with prosocial behavior score. Table 4 shows that compared with those without exposure, adolescents exposed to premigration traumatic events (adjusted OR [aOR], 2.49 [95% CI, 1.10-5.63]), who were treated unfairly (aOR, 3.77 [95% CI, 1.60-8.91]), and who faced English language barriers (aOR, 6.41 [95% CI, 1.98-20.79]) after resettlement were at a higher risk for PTSD.

**Table 3.** Factors Across Multiple Domains Associated With Strengths and Difficulties Questionnaire Prosocial Behavior Among Refugee Children and Adolescents in the Building a New Life in Australia Project

Factor	Strengths and Difficulties Questionnaire prosocial behavior, age category					
	Children, 5-10 y (n = 220)			Adolescents, 11-17 y (n = 412)		
	Unstandardized β coefficient (95% CI)^a^	*P* value	Standardized β coefficient	Unstandardized β coefficient (95% CI)^a^	*P* value	Standardized β coefficient
**Individual domain**						
Child level						
Age (1-y increase)^b^	0.03 (−0.18 to 0.25)	.75	0.02	−0.03 (−0.14 to 0.08)	.61	−0.03
Sex (reference category is girls)^b^	0.47 (−0.48 to 1.43)	.33	0.07	−0.17 (−0.60 to 0.26)	.44	−0.04
Premigration stressors^c^						
Exposed to traumatic events (reference category is no)	−1.00 (−2.11 to 0.10)	.07	−0.10	−0.48 (−0.98 to 0.01)	.06	−0.10
Safety or life badly threatened (reference category is no)	−0.42 (−1.47 to 0.63)	.43	−0.04	NA	NA	NA
English language barriers (reference category is no)^b^	−3.15 (−6.10 to −0.20)	.04	−0.23^d^	−0.55 (−1.21 to 0.11)	.10	−0.09
Rating of physical health (1-U increase)^b^	0.52 (−0.10 to 1.14)	.10	0.14	NA	NA	NA
Physical activity in past week (1-d increase)^b^	0.13 (−0.03 to 0.30)	.12	0.12	NA	NA	NA
Caregiver level						
Geographic region of caregiver’s birth^e^						
North Africa	1 [Reference]	NA	NA	NA	NA	NA
Middle East	−0.97 (−2.03 to 0.09)	.07	−0.13	NA	NA	NA
Southeast Asia (only Myanmar)	−0.94 (−4.33 to 2.45)	.58	−0.06	NA	NA	NA
Southern Asia	−1.89 (−3.56 to −0.22)	.03	−0.20^d^	NA	NA	NA
Central Asia (only Afghanistan)	−1.40 (−3.19 to 0.39)	.12	−0.15	NA	NA	NA
Sub-Saharan Africa	−1.64 (−4.21 to 0.94)	.21	−0.08	NA	NA	NA
Caregiver’s postmigration stressors^e^						
No. of social integration stressors (1-U increase)	NA	NA	NA	−0.40 (−0.74 to 0.06)	.02	−0.14^d^
**Family domain**						
Family structure (reference category is single)^e^	NA	NA	NA	0.62 (0.11 to 1.14)	.02	0.15^d^
Parenting warmth (1-U increase)^e^	0.08 (−0.03 to 0.19)	.17	0.13	NA	NA	NA
**School domain**						
Achievement award in last year (reference category is no)^c^	NA	NA	NA	0.21 (−0.29 to 0.71)	.41	0.05
**Community domain**						
Extracurricular engagement (reference category is no)^c^	NA	NA	NA	0.42 (−0.30 to 1.13)	.25	0.07
Neighborhood friendliness (reference category is disagree)^e^	NA	NA	NA	1.30 (0.22 to 2.38)	.02	0.04^d^

Abbreviation: NA, not available or applicable.

^a^ Weighted multivariable multilevel linear regression models were performed in which the migrating unit was the level 1 unit and factors in multiple domains were level 2 units.

^b^ Information came from both caregiver and child reports; caregiver-reported information was used for children aged 5 to 10 years, and adolescent-reported information used for adolescents aged 11 to 17 years.

^c^ Indicates child-reported information.

^d^ Significant standardized regression coefficients were ranked from the first to the second or third in each model.

^e^ Indicates caregiver-reported information.

**Table 4.** Factors Across Multiple Domains Associated With Posttraumatic Stress Disorder (PTSD) Among Refugee Adolescents in the Building a New Life in Australia Project

Factor	PTSD in adolescents aged 11-17 y (n = 412)	
	aOR (95% CI)^a^	*P* value
**Individual domain**		
Child level		
Age (1-y increase)^b^	0.80 (0.66 to 0.96)	.02
Sex (reference category is girls)^b^	1.03 (0.51 to 2.11)	.94
Premigration stressors^c^		
Exposed to traumatic events (reference category is no)	2.49 (1.10 to 5.63)	.03
Treated unfairly in last 6 mo (reference category is no)^c^	3.77 (1.60 to 8.91)	.002
English language barriers (reference category is no)^b^	6.41 (1.98 to 20.79)	.002
Caregiver level		
Geographic region of caregiver’s birth^d^		
North Africa	1 [Reference]	NA
Middle East	0.18 (0.02 to 1.78)	.14
Southeast Asia (only Myanmar)	NA	NA
Southern Asia	0.34 (0.03 to 3.82)	.38
Central Asia (only Afghanistan)	0.13 (0.01 to 1.41)	.09
Sub-Saharan Africa	NA	NA

Abbreviations: aOR, adjusted odds ratio; NA, not available or applicable.

^a^ Weighted multivariable multilevel regression models were performed in which the migrating unit was the level 1 unit and factors in multiple domains were level 2 units.

^b^ Information was from both caregiver and adolescent reports. If responses from both caregiver and adolescent reports were available, then self-reported data from the adolescents were used. If data were available from adolescent self-reports only or caregiver reports only, the available information was analyzed.

^c^ Indicates adolescent-reported information.

^d^ Indicates caregiver-reported information.

## Discussion

This study is one of the first to explore the premigration and postmigration multidomain factors associated with the psychosocial health of refugee children and adolescents after resettlement. Consistent with previous evidence related to refugees or similar situations,^31^ we observed that a higher proportion of refugee children and adolescents reported experiencing premigration potentially traumatic events, especially adolescents. In addition, this study found that these refugee children and adolescents may adapt to their resettled lives to some extent, report high levels of physical health, become accustomed to using English, and experience good parenting or good school activity engagements. However, our findings also demonstrated that emotional and behavioral problems among refugees aged 5 to 10 years and these problems and PTSD among refugees aged 11 to 17 years were relatively more common than in general adolescents,^5,32^ and these psychosocial health problems are also a sign of lower psychosocial adjustment and should not be ignored.

After incorporating all potentially significant factors from multiple domains, we found that exposure to premigration events and having family conflicts after resettlement were significantly associated with a higher SDQ total difficulties score among refugee children aged 5 to 10 years. Similarly, Gormez et al^33^ reported that exposure to cruelty or torture was significantly associated with the development of mental health problems among forcibly displaced Syrian children in Turkey; Emegwa and Saboonchi^34^ observed that family conflicts had negative effects on refugees’ subjective well-being. Notably, our study also observed positive associations of being treated unfairly and parenting harshness after resettlement with SDQ total difficulties for refugee adolescents aged 11 to 17 years, a result that was slightly different from the aforementioned associated factors among children aged 5 to 10 years, except for the family-domain factors. These findings were consistent with previous evidence^35^ that reported harsh parenting and perceived discrimination as having deleterious effects on the mental health of immigrant and refugee youth in Canada. A possible explanation might be that children and adolescents might have different vulnerabilities and protective systems affecting their resilience,^20^ and the factors associated with their mental health may vary. Nevertheless, for both child and adolescent refugees, the family was usually the primary source of support, which may enhance the adaptation of the children’s new life in their host countries. In contrast, a poor family environment or relationship may play an important role in the psychosocial ill health of refugee children and adolescents.^15^ Moreover, consistent with previous research,^36^ the present study also found that good school achievement or engagement was negatively associated with SDQ total difficulties among refugee children and adolescents. These results might be explained by the fact that apart from family, school is the primary living environment and source of support for refugee children and adolescents and plays a vital role in enhancing psychosocial adaptation and well-being in resettled young refugees.^36^

In addition, we found that for refugee children aged 5 to 10 years, facing English language barriers was negatively associated with their prosocial behavior score. A potential explanation for this finding is that children with language difficulties are at a higher risk of social exclusion and emotional and behavioral difficulties,^37^ particularly for younger refugee children who began a new life in their host countries. A language barrier can make them unable or unwilling to communicate with others or be involved in local social activities, which is damaging to developing prosocial behaviors.^38^ Although this study did not observe a significant association between having English language barriers and prosocial behavior for refugee adolescents aged 11 to 17 years, we found that the number of social integration stressors was negatively associated with prosocial behavior among them. This finding may be explained by the fact that although refugee adolescents may have learned English well before migration, they may experience complex social integration stressors (eg, sociocultural maladaptation), which may affect their communication and interpersonal skills.^39^

In the BNLA study, PTSD was only assessed among refugee adolescents aged 11 to 17 years. In line with previous evidence on PTSD in migrants,^7,40^ this study found that after incorporating all potentially multidomain factors associated with PTSD, only exposure to premigration traumatic events and postmigration stressors (ie, being treated unfairly and facing English language barriers after resettlement) were positively associated with the presence of PTSD. Adolescent PTSD is a debilitating mental illness that may differ from experiencing emotional and behavioral difficulties.^41^ Professional interventions, including screening, making appropriate diagnoses, and providing treatment, are recommended for those with positive findings for PTSD to promote mental health among refugee adolescents, particularly those experiencing premigration traumatic events or postmigration discrimination.

### Limitations

There are several limitations to this study. First, based on the selection standard in the child module of the BNLA cohort, there were no unaccompanied children in the sample, and psychosocial problems may be more common in those children. Second, using the self-report method to collect data may subject our findings to recall bias. Third, some information used in this study was provided by caregivers or adolescents, who are not completely interchangeable. Fourth, PTSD was only measured among refugees aged 11 to 17 years. However, the traumatic illness of younger refugee children cannot be ignored, and appropriate measurements are suggested in future surveys. Fifth, because the BNLA project was initiated before the publication of the *Diagnostic and Statistical Manual of Mental Disorders (Fifth Edition)*, the scales used in the project are based on *DSM-IV*. Sixth, the small sample size (relevant primarily for PTSD) may be a limitation of this study. Seventh, causal associations cannot be defined because of the cross-sectional nature of the study data.

## Conclusions

In this cross-sectional study of refugee children and adolescents, we observed some discrepancies in the associations of the individual-, family-, school-, and community-level factors with psychosocial health after resettlement between refugee children aged 5 to 10 years and adolescents aged 11 to 17 years. However, for both age groups, premigration traumatic experiences and several postmigration family- and school-related factors and social integration factors (eg, conflicting family environment, being treated unfairly, or having language barriers) were important correlates of refugees’ psychosocial health after resettlement. Accordingly, the findings suggest that strengthening family- and school-centered psychosocial care and social integration programs targeting related stressors may have potential value in improving the psychosocial health of refugee children and adolescents after resettlement. However, future randomized clinical trials are needed to confirm these conclusions.

## Notes

Supplement 1. **eMethods 1.** The Detailed Process of Participant Selection **eMethods 2.** Details on the Definition and Code of These Independent and Dependent Variables **eFigure.** Categories of Different Domains and Psychosocial Health **eTable 1.** Emotional and Behavioral Health and PTSD Among Young Refugees in the Building a New Life in Australia Project **eTable 2.** Associations Between Factors in Multiple Domains and SDQ Total Difficulties: Weighted Univariable Multilevel Linear Regression Models **eTable 3.** Associations Between Factors in Multiple Domains and SDQ Prosocial Behavior: Weighted Univariable Multilevel Linear Regression Models **eTable 4.** Associations Between Factors in Multiple Domains and PTSD: Weighted Univariable Multilevel Logistic Regression Models
eReferences

Supplement 2.
Data Sharing Statement
