# Supplementary material for: Characterization of Premigration and Postmigration Multidomain Factors and Psychosocial Health Among Refugee Children and Adolescents After Resettlement in Australia
Source: JAMA Netw Open. 2023 Apr 6;6(4):e235841. doi: 10.1001/jamanetworkopen.2023.5841 (PMC10080375; doi:10.1001/jamanetworkopen.2023.5841)
Supplement: Supplement 1. — eMethods 1. The Detailed Process of Participant Selection eMethods 2. Details on the Definition and Code of These Independent and Dependent Variables eFigure. Categories of Different Domains and Psychosocial Health eTable 1. Emotional and Behavioral Health and PTSD Among Young Refugees in the Building a New Life in Australia Project eTable 2. Associations Between Factors in Multiple Domains and SDQ Total Difficulties: Weighted Univariable Multilevel Linear Regression Models eTable 3. Associations Between Factors in Multiple Domains and SDQ Prosocial Behavior: Weighted Univariable Multilevel Linear Regression Models eTable 4. Associations Between Factors in Multiple Domains and PTSD: Weighted Univariable Multilevel Logistic Regression Models eReferences [file jamanetwopen-e235841-s001.pdf]

Supplementary Online Content

Guo L, Li L, Xu K, et al. Characterization of premigration and postmigration multidomain factors and psychosocial health among refugee children and adolescents after resettlement in Australia. *JAMA Netw Open*. 2023;6(4):e235841. doi:10.1001/jamanetworkopen.2023.5841

eMethods 1. The Detailed Process of Participant Selection
eMethods 2. Details on the Definition and Code of These Independent and Dependent Variables
eFigure. Categories of Different Domains and Psychosocial Health
eTable 1. Emotional and Behavioral Health and PTSD Among Young Refugees in the Building a New Life in Australia Project
eTable 2. Associations Between Factors in Multiple Domains and SDQ Total Difficulties: Weighted Univariable Multilevel Linear Regression Models
eTable 3. Associations Between Factors in Multiple Domains and SDQ Prosocial Behavior: Weighted Univariable Multilevel Linear Regression Models
eTable 4. Associations Between Factors in Multiple Domains and PTSD: Weighted Univariable Multilevel Logistic Regression Models
eReferences

This supplementary material has been provided by the authors to give readers additional information about their work.

© 2023 Guo L et al. *JAMA Network Open*.

eMethods 1. The Detailed Process of Participant Selection

Study population and sampling

The Building a New Life in Australia (BNLA) study is a longitudinal national refugee-based cohort study that traced the settlement journey of recently resettled humanitarian migrants in Australia over five waves (2013-2018) and investigated outcomes and risk factors related to this process [1,2]. BNLA participants were recruited from 11 Australian sites covering major cities and regional areas (Sydney, Brisbane, Melbourne, Adelaide, Perth, and six other cities), with the highest number of humanitarian migrants settling between November 2010 and October 2011. Details about the BNLA study sampling and follow-up procedures have been published elsewhere [1]. To date, five waves of data have been completed, with data collected in Waves 1, 3, and 5 through a computer-assisted self-interview and in Waves 2 and 4 through a computer-assisted telephone interview [2]. Participants in the BNLA study consisted of "principal" and "secondary" applicants for a humanitarian migrant visa in Australia granted in the period preceding the study. Principal applicants were the lead applicants within a migrating unit (typically a family) named on the visa application. Secondary applicants were other members of the migrating unit (including children and spouses) residing with the principal applicant and had to be aged 15 years or older. During the initial recruitment stage, the eligibility criteria for the principal applicant were (1) aged 18 years or older, (2) as a "principal applicant" for a humanitarian visa that was granted 3 to 6 months prior to the first wave survey of the BNLA project (i.e., May to December 2013) and already holding a permanent protection visa (the "offshore group"), or granted a permanent protection visa in the prior 3 to 6 months after arrival in Australia by boat or on another visa type such as a student or tourist visa (the "onshore" group). From the settlement database, a total of 4035 principal applicants were identified as potential participants, and 2031 were successfully contacted after initial contact (the BNLA user guide does not provide the details or criteria for contacting 2769 of the total 4035 applicants and not contacting the other 1266 applicants), and 1509 principal applicants completed a survey in Wave 1. Besides, 755 adult secondary applicants and 135 secondary adolescent applicants who were in the same visa application and aged 15 years or older were also recruited to Wave 1, yielding 2399 participants in all [3]. During the follow-up, initial contact through a primary approach letter was addressed to all participants who completed the Wave 1 survey and did not subsequently withdraw from the study. Through this letter, interviewers would arrange appointments with participants to conduct interviews.

Wave 3 data were collected between October 2015 and February 2016 and included interviews with 1155 principal applicants and 739 secondary applicants. Notably, a child

© 2023 Guo L et al. *JAMA Network Open*.

module mainly about children's settlement experiences and social and emotional well-being was first introduced in the BNLA project. Wave 3 survey was the first time so far in a BNLA study that included a child module targeting children and adolescents aged 5 to 17 years in the migrating unit as a nested component of the broader study [1,2]. Initial sampling for the child module occurred by randomly selecting up to two children aged 5 to 17 years in each household. In households with multiple children but only one child aged 11 to 17 years, the eldest child was recruited, and one younger child aged 5 to 10 years was randomly selected. In households with only younger children, two children aged 5 to 10 years were randomly selected. The caregivers of children aged 5 to 10 years were invited to complete the child module, which was administered via pencil and paper. Adolescents aged 11 to 17 years and their caregivers were invited to complete the child module. Based on the selection standard, there were no unaccompanied children in the sample.

In this study, at first, there were two components to the child module: the first collected information about 279 children aged 5 to 10 years from their primary caregivers; the second collected information from 415 older children and adolescents aged 11 to 17 years themselves and their primary caregivers [2]; twelve children were excluded for only having their own responses. Next, after carefully examining the database, 59 of the 279 children aged 5 to 10 years were actually in the age group between 11 and 17 years, and 3 of the 415 older children and adolescents aged 11 to 17 years were actually in the age group between 5 and 10 years; then their information was also excluded for not meeting the selection standard. **Figure 1** describes the recruitment process of participants (principal and secondary applicants) in Wave 1 and the subsequent recruitment of caregivers, children, and adolescents in Wave 3.

© 2023 Guo L et al. *JAMA Network Open*.

eMethods 2. Details on the Definition and Code of These Independent and Dependent Variables

1. The child module

There were two components associated with the child module:

- The first component was a child module including questions for up to two of their children aged 5 to 17 years, which was completed by the primary caregiver (in most cases, mothers). The questions covered how the children were doing at school, whether they were making friends, their health status and well-being, how they were adjusting to life in Australia, and the Strengths and Difficulties Questionnaire (SDQ).
- The second component of the child module involved a child self-report questionnaire, which was fulfilled by children aged 11 to 17 years. The questionnaire included questions covering health status, trauma experience, antisocial behavior, physical activity, academic and sporting achievements and awards received, the Strengths and Difficulties Questionnaire (SDQ), and the Posttraumatic Stress Disorder-8 (PTSD-8) inventory. There was some cross-over in the type of content asked in each questionnaire, with the SDQ, health status, and trauma covered in both the parent-complete and child-complete questionnaires to allow comparison of critical outcomes for young people from the perspective of parents and the children themselves [2].

(1) The child module only reported by the primary caregivers

Parenting style

Parenting styles, including warmth and hostility, were assessed using caregiver self-rating separately [4]. Parenting warmth or parenting hostility each consists of five questions with the response given on a five-point Likert scale as follows: 1= never/almost never, 2= rarely, 3= sometimes, 4= often, and 5= always/almost always. Examples of warmth questions included: “How often do you have warm, close times together with this child?”; “How often do you enjoy listening to this child and doing things with him/her?”; “I feel that I am good at getting this child to do what I want him/her to do”. Examples of hostility questions included: “I have been angry with this child.”; “I have lost my temper with this child.”; “I have raised my voice or shouted at this child”.

Child school achievement and absenteeism

School achievement was measured by asking caregivers with children enrolled in school the question: “How would you describe [named child]’s overall achievement at school?” Responses included 1= excellent, 2= above average, 3= average, 4= below average, and 5= well below average. Caregiver responses to the open-ended question assessed school absenteeism: “During the previous four weeks of school, how many days has [named child]

© 2023 Guo L et al. *JAMA Network Open*.

been absent?

(2) The child module only reported by adolescents aged 11 to 17 years

School award

School award was assessed by asking children the question: “In the last year, have you won any awards or been recognized for doing well in certain activities?” Responses included (1) winning an academic award, (2) receiving a community service award, (3) being selected to represent the school, (4) receiving an award in sports, and (5) receiving an award in music, arts, dance performance or drama.

Engagement in extracurricular activities

Children aged 11 to 17 years were also asked about their engagement in extracurricular activities by the question: “In the last 6 months, have you regularly attended any of these activities?” Responses options included (1) individual sport (e.g., swimming or athletics), (2) team sport (e.g., football or netball), (3) musical instruments or singing, (4) ballet or other dance, and (5) religious group.

Treated unfairly

Being treated unfairly was measured by asking children the question: “In the last 6 months, have you been treated unfairly or badly because of your language or accent, skin color, religious beliefs, or cultural background? Responses were categorized into 1= yes and 2= no.

Posttraumatic stress disorder (PTSD)

PTSD was assessed using the PTSD-8 scale derived from the Harvard Trauma Questionnaire Part IV and has been validated and used cross-culturally [5]. The PTSD-8 covers all three symptom clusters of the DSM-IV-based PTSD diagnosis (i.e., intrusion, avoidance, and hyperarousal). The PTSD-8 consists of eight items, each addressing the frequency of specific symptoms of PTSD over the past week. Responses for each item are rated on a four-point Likert scale (1= not at all, 2= rarely, 3= sometimes, and 4= most of the time). The total score varied from 8 to 32. PTSD was determined to be present if each PTSD symptoms cluster had at least one item score of 3 (sometimes) or higher [5]. The PTSD-8 has been shown to have good psychometric properties in patients with whiplash, rape victims, and disaster victims (the Cronbach’s α=0·83, 0·84, 0.85 respectively) and high correlations with the Trauma Symptom Checklist, indicating construct validity [6,7]. In the present study, the internal consistency reliability of PTSD-8 was 0·96.

(3) The child module reported by both caregivers and adolescents aged 11 to 17 years

Health status

The child’s health status was assessed by asking the question: “In general, would you say [named child]’s/your health is (1) excellent, (2) very good, (3) good, (4) fair, or (5) poor?

© 2023 Guo L et al. *JAMA Network Open*.

The child's physical activity was measured by asking the open-ended question: "In the last 7 days, on how many days have [named child]/you done a total of 60 min or more of physical activity, which was enough to raise [named child]'s/your breathing rate?

Premigration stressors

Premigration stressors were measured, including children's experience of exposure to traumatic events and experience of safety- or life-threatening events. Children's experience of exposure to traumatic events was assessed by the question: "When families have been directly exposed to situations of war or fleeing their country, they may be exposed to traumatic events such as extreme living conditions, direct experience of combat, or forced separation from family. Has something like this happened to [named child]/you?" Responses were 1= yes and 2= no. Children's experience of safety- or life-threatening events was measured by the question: "In addition to the events described above, have [named child]/you ever had something else happened in which [named child]'s/your safety or life was badly threatened?" Responses included 1= yes and 2= no.

English language barriers

Having English language barriers was measured by this question: "How much do you agree or disagree with the following statements: [named child]/I often use English for communication (e.g., talking to friends/family, television, internet)?" Responses included 1= strongly disagree, 2= disagree, 3= neither disagree nor agree, 4= agree, and 5= strongly agree. Respondents who reported disagreeing or strongly disagreeing with this question were considered to have English language barriers.

Emotional and behavioral problems

Emotional and behavioral problems were assessed by the parent-report and self-report versions of the Strengths and Difficulties Questionnaire (SDQ), which have been validated and widely used with satisfactory psychometric properties (https://www.sdqinfo.org/a0.html) [24-26]. The SDQ comprises 25 items operationalizing five subscales: prosocial behavior, peer problems, emotional problems, conduct problems, and hyperactivity. Each subscale includes five items with the response given on a three-point Likert scale as follows: 1= not true, 2= somewhat true, and 3= certainly true. Examples of items of the parent-report version were: "considerate of other people's feelings (prosocial behavior item); "gets along better with adults than other children (peer problem item)"; "many worries, often seem worried (emotional problem item)"; "often fights with other children or bullies them (conduct problem item)"; "easily distracted, concentration wanders (hyperactivity item)". Examples of items of the self-report version were: 'I try to be nice to other people, and I care about their feelings' (prosocial behavior item); 'I am usually on my

© 2023 Guo L et al. *JAMA Network Open*.

own, and I generally play alone or keep to myself' (peer problem item); 'I get a lot of headaches, stomach-aches or sickness' (emotional symptom item); 'I usually do as I am told' (conduct problem inverse item); 'I think before I do things' (hyperactivity inverse item). Each of the five SDQ subscale scores ranged from 0 to 10. The total difficulties scores indicate the sum of the following four subscales (excepting prosocial behavior subscale): emotional problems, peer problems, conduct problems, and hyperactivity; higher scores represent a greater level of difficulties. In contrast, lower scores on the prosocial behavior subscale indicate greater psychological problems. In this study, the Cronbach's alpha for prosocial behavior was 0.76 in the present study; 0.75 for peer problems; 0.77 for emotional problems; 0.74 for conduct problems; 0.70 for hyperreactivity. Moreover, in this study, we also described the validated standard cut-off values for total difficulties and each subscale of SDQ, including "normal", "borderline", and "abnormal" [11].

Demographics, including the child's sex (1= male, 2= female) and age, were also provided by caregivers and adolescents aged 11 to 17 years.

2. Measures reported by caregivers in the Wave 3 survey

Country of caregiver's birth

The country of caregiver's birth included the middle east, central Asia (only Afghanistan), southern Asia, Southeast Asia (only Myanmar), sub-Saharan Africa, and North Africa.

Family structure

Family structure was defined based on information reported by the principal applicant, which identified the relationship of all household members to themselves (e.g., spouse, unrelated child, grandchild, biological child). This allowed for a classification of family structure in terms of whether the principal applicant was in a couple or single, and whether other family members lived in the household.

Time between arrival in Australia and an interview date

The time between arrival in Australia and an interview date was categorized into (1) 1-2 years; (2) 2-3 years; (3) ≥ 3 years.

Caregiver's postmigration stressors

A total of 12 questions related to postmigration-related or resettlement-related stressors (responses including yes or no) were asked to caregivers. In this study, these questions were categorized into seven subgroups: the number of economic stressors (including working, house, and financial situations); the number of concerns about their family in Australia (including caring for family/your family's health/family's safety); discrimination (yes or no); loneliness (yes or no); family conflicts in Australia (including conflict/tension with partner and conflict/tension with children; responses were categorized into yes or no); problems with

adjustment to life in Australia (yes or no); having English language barriers (yes or no).

Ethnic or religious community support

Ethnic or religious community support was measured by asking the caregiver the following question: "do you feel that you have been given support/comfort in Australia from your national or ethnic community, your religious community, or other community groups?" Responses included 1= yes, 2= sometimes, and 3= no.

Neighborhood friendliness/safety

Neighborhood friendliness/safety was assessed by asking the caregiver to provide a response to the following statement about their neighborhood (local area): (1)The people in my neighborhood are friendly; (2) I feel safe in my neighborhood. Responses were categorized into 1= strongly agree, 2= agree, 3= disagree, and 4= strongly agree.

© 2023 Guo L et al. *JAMA Network Open*.

eFigure. Categories of Different Domains and Psychosocial Health

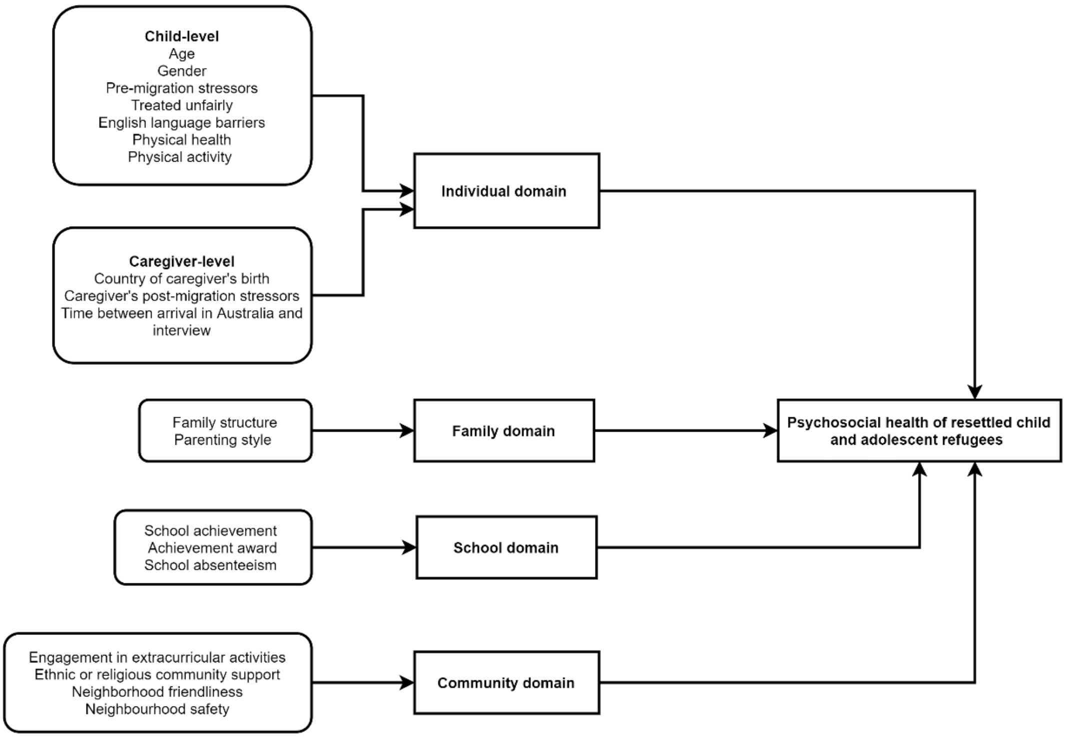

© 2023 Guo L et al. *JAMA Network Open*.

eTable 1. Emotional and Behavioral Health and PTSD Among Young Refugees in the Building a New Life in Australia Project

	Age category	
	**5-10 years (n=220)**	**11-17 years (n=412)**
SDQ score, mean (SD) [a]		
Total difficulties	8.6 (6.5)	8.7 (5.6)
Peer problems	2.0 (2.2)	2.3 (1.8)
Hyperactivity/inattention	2.9 (2.8)	2.5 (1.8)
Conduct problems	1.2 (2.1)	1.4 (1.6)
Emotional problems	1.8 (2.7)	2.6 (2.3)
Prosocial behavior	7.2 (3.6)	8.0 (1.9)
SDQ score [b]		
Total difficulties		
Normal (0-13)	180 (87.4)	356 (88.1)
Borderline (14-16)	11 (5.3)	35 (8.7)
Abnormal (17-40)	15 (7.3)	13 (3.2)
Peer problems		
Normal (0-2)	156 (75.7)	311 (76.2)
Borderline (3)	39 (18.9)	79 (19.4)
Abnormal (4-10)	11 (5.3)	18 (4.4)
Hyperactivity/inattention		
Normal (0-5)	174 (84.5)	391 (95.8)
Borderline (6)	15 (7.3)	9 (2.2)
Abnormal (7-10)	17 (8.3)	8 (2.0)
Conduct problems		
Normal (0-2)	181 (87.9)	365 (89.2)
Borderline (3)	14 (6.8)	23 (5.6)
Abnormal (4-10)	11 (5.3)	21 (5.1)
Emotional problems		
Normal (0-3)	190 (92.2)	363 (88.8)
Borderline (4)	2 (1.0)	21 (5.1)
Abnormal (5-10)	14 (6.8)	25 (6.1)
Prosocial behavior		
Normal (6-10)	178 (86.4)	378 (92.2)
Borderline (5)	12 (5.8)	16 (3.9)
Abnormal (0-4)	16 (7.8)	16 (3.9)
PTSD (yes) [b]	-	59 (14.3)

SDQ=Strengths and Difficulties Questionnaire; PTSD=posttraumatic stress disorder.

[a]: Data were presented as mean (standard deviation).

[b]: Data were reported in the form of the number (%). Cut-off values for total difficulties and each subscale of SDQ were presented within parentheses.

© 2023 Guo L et al. *JAMA Network Open*.

eTable 2. Associations Between Factors in Multiple Domains and SDQ Total Difficulties: Weighted Univariable Multilevel Linear Regression Models

	**SDQ total difficulties**			
	**Age category**			
	**5-10 years (n=220)**		**11-17 years (n=412)**	
	$\beta_{unstand}$ **(95% CI)***	***P* value**	$\beta_{unstand}$ **(95% CI)***	***P* value**
**Individual domain**				
Child-level				
Age (1-year increase) [c]	0.30 (-0.26~0.86)	.29	0.28 (-0.01~0.57)	.06
Sex (ref.=girl) [c]	0.83 (-1.26~2.92)	.44	-1.51 (-2.72~-0.31)	.02
Premigration stressors [a]				
Exposed to traumatic events (ref.=no)	2.60 (0.60~4.60)	.01	1.34 (-0.16~2.85)	.08
Safety or life badly threatened (ref.=no)	0.27 (-1.87~2.42)	.80	1.48 (-0.21~3.17)	.09
Treated unfairly in last 6 months (ref.=no) [a]	NR		3.76 (2.08~5.43)	<0.001
English language barriers (ref.=no) [c]	3.53 (-3.23~10.29)	.30	0.55 (-1.42~2.52)	.59
Rating of physical health (1-unit increase) [c]	-2.50 (-3.69~-1.33)	<0.001	-1.43 (-2.09~-0.77)	<0.001
Physical activity in past week (1-day increase) [c]	0.28 (0.05~0.51)	.02	-0.10 (-0.28~0.08)	.27
Caregiver-level				
Country of caregiver's birth (ref.= North Africa) [b]				
Middle east	1.29 (-2.30~4.89)	.48	-0.33 (-1.69~1.02)	.63
Southeast Asia (only Myanmar)	0.44 (-6.72~7.60)	.90	-1.41 (-3.89~1.06)	.26
Southern Asia	3.22 (-0.30~6.74)	.07	-0.86 (-3.65~1.92)	.54
Central Asia (only Afghanistan)	3.93 (2.00~5.85)	<0.001	-2.88 (-6.00~0.25)	.07
Sub-Saharan Africa	3.15 (1.48~4.82)	<0.001	-2.69 (-3.88~-1.49)	<0.001
Caregiver's postmigration stressors [b]				
Number of economic stressors (1-unit increase)	0.77 (-0.22~1.77)	.13	0.22 (-0.29~0.73)	.40
Number of concerns about family in Australia (1-unit increase)	0.54 (-0.73~1.82)	.40	0.71 (-0.13~1.55)	.10
Number of social integration stressors (1-unit increase)	0.92 (-0.79~2.63)	.29	0.37 (-0.55~1.29)	.43
Discrimination (ref.=no)	-2.92 (-7.45~1.61)	.21	1.48 (-1.43~4.40)	.32
Loneliness (ref.=no)	1.10 (-1.94~4.14)	.48	1.00 (-0.61~2.61)	.23
Family conflicts in Australia (ref.=no)	5.41 (-0.55~11.36)	.08	0.28 (-2.00~2.57)	.81
Problems with adjustment to life in Australia (ref.=no)	0.80 (-2.08~3.68)	.59	1.05 (-0.66~2.77)	.23
Time between arrival in Australia and interview (ref.=1-2 years) [b]				
2-3 years	0.12 (-3.18~3.41)	.94	4.16 (-0.36~8.68)	.07

© 2023 Guo L et al. *JAMA Network Open*.

eTable 2. Associations between factors in multiple domains and SDQ total difficulties: weighted univariable multilevel linear regression models (continued)

≥3 years	-2.26 (-7.85~3.34)	.43	2.18 (-2.79~7.14)	.39
**Family domain**				
Family structure (ref.=single) [b]	0.51 (-2.39~3.41)	.73	-0.47 (-1.94~1.01)	.53
Parenting style [b]				
Parenting warmth (1-unit increase)	-0.04 (-0.22~0.13)	.64	-0.003 (-0.109~0.104)	.96
Parenting harshness (1-unit increase)	0.41 (0.17~0.65)	0.001	0.17 (0.06~0.27)	0.002
**School domain**				
Achievement award in last year (ref.=no) [a]	NR		0.11 (-1.25~1.45)	.88
School achievement average or above average (ref.=no) [b]	-3.34 (-6.59~-0.08)	.04	-2.61 (-5.74~0.52)	.10
School absenteeism (1-day increase) [b]	0.77 (0.16~1.39)	.01	0.50 (0.25~0.74)	<0.001
**Community domain**				
Extracurricular engagement (ref.=no) [a]	NR		-2.17 (-4.73~0.38)	.10
Ethnic or religious community support (ref.=no) [b]				
Sometimes	-1.39 (-3.91~1.14)	.28	-0.54 (-2.39~1.32)	.57
Yes	-3.22 (-5.57~-0.86)	0.008	-0.13 (-1.41~1.16)	.85
Neighborhood friendliness (ref.=disagree) [b]	-4.28 (-7.76~-0.80)	.02	-1.23 (-4.66~2.19)	.48
Neighborhood safety (ref.=disagree) [b]	-4.14 (-6.87~-1.41)	0.003	-3.34 (-6.06~-0.62)	.02

NR= not reported; $\beta_{unstand}$=unstandardized regression coefficient; 95% CI=95% confidence interval; ref.=reference; SDQ=Strengths and Difficulties Questionnaire.

[a]: Child-reported information.

[b]: Caregiver-reported information.

[c]: Information from both caregiver and child reports, caregiver-reported information used for children aged 5 to 10 years, and child-reported information used for children aged 11 to 17 years.

[*]: Univariable multilevel logistic regression models were performed, in which the migrating unit was the level 1 unit and factors in multiple domains were level 2 units.

© 2023 Guo L et al. *JAMA Network Open*.

eTable 3. Associations Between Factors in Multiple Domains and SDQ Prosocial Behavior: Weighted Univariable Multilevel Linear Regression Models

	**SDQ prosocial behavior**			
	**Age category**			
	**5-10 years (n=220)**		**11-17 years (n=412)**	
	$\beta_{unstand}$ **(95% CI)***	***P* value**	$\beta_{unstand}$ **(95% CI)***	***P* value**
**Individual domain**				
Child-level				
Age (1-year increase) [c]	0.08 (-0.17~0.33)	.54	-0.07 (-0.17~0.04)	.21
Sex (ref.=girl) [c]	-0.85 (-1.89~0.18)	.11	-0.35 (-0.79~0.09)	.12
Premigration stressors [a]				
Exposed to traumatic events (ref.=no)	-1.14 (-2.16~-0.12)	.03	0.42 (-0.02~0.86)	.06
Safety or life badly threatened (ref.=no)	-1.32 (-2.31~-0.32)	.01	0.37 (-0.10~0.85)	.12
Treated unfairly in last 6 months (ref.=no) [a]	NR		-0.53 (-1.28~0.23)	.17
English language barriers (ref.=no) [c]	-3.67 (-6.31~-1.02)	0.007	-0.60 (-1.20~0.01)	.05
Rating of physical health (1-unit increase) [c]	0.77 (0.21~1.33)	0.007	0.14 (-0.07~0.36)	.20
Physical activity in past week (1-day increase) [c]	0.19 (0.04~0.34)	.02	0.06 (-0.02~0.14)	.14
Caregiver-level				
Country of caregiver's birth (ref.= North Africa) [b]				
Middle east	-1.62 (-2.40~-0.84)	<0.001	-0.08 (-1.49~1.34)	.92
Southeast Asia (only Myanmar)	-3.71 (-5.96~-1.46)	0.001	-0.55 (-2.23~1.12)	.52
Southern Asia	-2.76 (-4.69~-0.84)	0.005	-0.37 (-1.89~1.15)	.64
Central Asia (only Afghanistan)	-2.71 (-4.07~-1.36)	<0.001	-0.43 (-1.86~1.00)	.56
Sub-Saharan Africa	-2.53 (-4.80~-0.26)	.03	-0.95 (-3.93~2.02)	.53
Caregiver's postmigration stressors [b]				
Number of economic stressors (1-unit increase)	0.23 (-0.22~0.69)	.31	0.06 (-0.12~0.23)	.52
Number of concerns about family in Australia (1-unit increase)	0.29 (-0.35~0.92)	.37	0.08 (-0.23~0.39)	.63
Number of social integration stressors (1-unit increase)	0.39 (-0.45~1.23)	.36	0.38 (-0.03~0.78)	.07
Discrimination (ref.=no)	-1.97 (-5.48~1.54)	.27	-0.61 (-1.69~0.47)	.27
Loneliness (ref.=no)	0.23 (-1.27~1.73)	.76	-0.11 (-0.66~0.43)	.69
Family conflicts in Australia (ref.=no)	-0.57 (-3.20~2.06)	.67	-0.33 (-1.24~0.57)	.47
Problems with adjustment to life in Australia (ref.=no)	-1.17 (-2.81~0.47)	.16	0.10 (-0.49~0.68)	.75

© 2023 Guo L et al. *JAMA Network Open*.

eTable 3. Associations between factors in multiple domains and SDQ prosocial behavior: weighted univariable multilevel linear regression models (continued)

Time between arrival in Australia and interview (ref.=1-2 years) [b]				
2-3 years	0.39 (-1.91~2.69)	.74	1.07 (-1.27~3.40)	.37
≥3 years	-0.16 (-1.75~1.43)	.84	0.42 (-1.72~2.56)	.70
**Family domain**				
Family structure (ref.=single) [b]	0.25 (-1.31~1.82)	.75	0.80 (0.14~1.46)	.02
Parenting style [b]				
Parenting warmth (1-unit increase)	0.14 (0.05~0.23)	0.002	0.02 (-0.02~0.05)	.32
Parenting harshness (1-unit increase)	0.08 (-0.04~0.21)	.18	0.03 (-0.02~0.07)	.21
**School domain**				
Achievement award in last year (ref.=no) [a]	NR		0.54 (-0.03~1.11)	.06
School achievement average or above average (ref.=no) [b]	0.69 (-0.88~2.26)	.39	0.11 (-0.42~0.64)	.69
School absenteeism (1-day increase) [b]	-0.10 (-0.22~0.20)	.93	-0.07 (-0.17~0.04)	.22
**Community domain**				
Extracurricular engagement (ref.=no) [a]	NR		1.08 (0.02~2.13)	.05
Ethnic or religious community support (ref.=no) [b]				
Sometimes	0.46 (-0.63~1.55)	.41	-0.20 (-0.65~0.26)	.40
Yes	0.04 (-1.21~1.28)	.95	0.11 (-0.72~0.93)	.80
Neighborhood friendliness (ref.=disagree) [b]	-1.89 (-5.36~1.58)	.28	0.97 (0.03~1.92)	.04
Neighborhood safety (ref.=disagree) [b]	-0.53 (-3.42~2.37)	.72	0.19 (-1.11~1.50)	.77

NR=not reported; $\beta_{unstand}$=unstandardized regression coefficient; 95% CI=95% confidence interval; ref.=reference; SDQ=Strengths and Difficulties Questionnaire.

[a]: Child-reported information.

[b]: Caregiver-reported information.

[c]: Information from both caregiver and child reports, caregiver-reported information used for children aged 5 to 10 years, and child-reported information used for children aged 11 to 17 years.

[*]: Univariable multilevel logistic regression models were performed, in which the migrating unit was the level 1 unit and factors in multiple domains were level 2 units.

© 2023 Guo L et al. *JAMA Network Open*.

eTable 4. Associations Between Factors in Multiple Domains and PTSD: Weighted Univariable Multilevel Logistic Regression Models

	**PTSD**	
	**Age category**	
	**11-17 years (n=412)**	
	**OR (95% CI)***	***P* value**
**Individual domain**		
Child-level		
Age (1-year increase) [c]	0.90 (0.78~1.04)	.17
Sex (ref.=girl) [c]	1.13 (0.57~2.22)	.73
Premigration stressors [a]		
Exposed to traumatic events (ref.=no)	2.57 (1.25~5.28)	.01
Safety or life badly threatened (ref.=no)	1.82 (0.84~3.95)	.13
Treated unfairly in last 6 months (ref.=no) [a]	4.11 (2.04~8.30)	<0.001
English language barriers (ref.=no) [c]	3.26 (1.11~9.53)	.03
Rating of physical health (1-unit increase) [c]	1.13 (0.83~1.54)	.45
Physical activity in past week (1-day increase) [c]	1.05 (0.96~1.16)	.29
Caregiver-level		
Country of caregiver's birth (ref.= North Africa) [b]		
Middle east	0.29 (0.03~2.20)	.24
Southeast Asia (only Myanmar)	1	
Southern Asia	0.35 (0.03~3.62)	.38
Central Asia (only Afghanistan)	0.18 (0.02~1.38)	.10
Sub-Saharan Africa	0.18 (0.01~3.18)	.24
Caregiver's postmigration stressors [b]		
Number of economic stressors (1-unit increase)	1.18 (0.90~1.55)	.24
Number of concerns about family in Australia (1-unit increase)	1.09 (0.69~1.72)	.70
Number of social integration stressors (1-unit increase)	0.93 (0.56~1.56)	.79
Discrimination (ref.=no)	1.67 (0.42~6.65)	.47
Loneliness (ref.=no)	1.27 (0.53~3.03)	.59
Family conflicts in Australia (ref.=no)	1.33 (0.41~4.31)	.63
Problems with adjustment to life in Australia (ref.=no)	1.58 (0.72~3.44)	.25
Time between arrival in Australia and interview (ref.=1-2 years) [b]		
2-3 years	0.57 (0.07~4.95)	.61
≥3 years	0.52 (0.03~10.69)	.68
**Family domain**		
Family structure (ref.=single) [b]	0.89 (0.41~1.90)	.76
Parenting style [b]		
Parenting warmth (1-unit increase)	1.06 (0.99~1.13)	.12
Parenting harshness (1-unit increase)	1.01 (0.96~1.07)	.59
**School domain**		
Achievement award in last year (ref.=no) [a]	0.96 (0.47~1.98)	.91
School achievement average or above average (ref.=no) [b]	0.84 (0.23~3.09)	.79
School absenteeism (1-day increase) [b]	1.00 (0.87~1.15)	.98
**Community domain**		
Extracurricular engagement (ref.=no) [a]	0.77 (0.28~2.10)	.60
Ethnic or religious community support (ref.=no) [b]		
Sometimes	1.18 (0.48~2.90)	.73
Yes	0.73 (0.32~1.63)	.44
Neighborhood friendliness (ref.=disagree) [b]	0.49 (0.12~1.98)	.31
Neighborhood safety (ref.=disagree) [b]	0.40 (0.05~3.17)	.38

$\beta_{unstand}$=unstandardized regression coefficient; ref.=reference; PTSD=posttraumatic stress disorder; OR=odds ratio; 95% CI= 95% confidence interval.

[a]: Child-reported information.

[b]: Caregiver-reported information.

[c]: Information from both caregiver and child reports, and caregiver-reported information used for children aged 5 to 10 years, child-reported information used for children aged 11 to 17 years.

[*]: Univariable multilevel logistic regression models were performed, in which the migrating unit was the level 1 unit and factors in multiple domains were level 2 units.

© 2023 Guo L et al. *JAMA Network Open*.

eReferences

1. Edwards B, Smart D, De Maio J, Silbert M, Jenkinson R. Cohort Profile: Building a New Life in Australia (BNLA): the longitudinal study of humanitarian migrants. *Int J Epidemiol*. 2018;47(1):20-20h. doi:10.1093/ije/dyx218

2. Rioseco P, De Maio J, Hoang C. The Building a New Life in Australia (BNLA) Dataset: A Longitudinal Study of Humanitarian Migrants in Australia. *The Australian Economic Review*. 2017;50(3):356-362.

3. Australian Department Of Social Services. Building a New Life in Australia: The Longitudinal Study of Humanitarian Migrants: Data Users Guide Release 5.0: Australian Institute of Family Studies, Colmar Brunton Social Research, 2019.

4. Edwards B, Gray M, Wise S, et al. Early impacts of Communities for Children on children and families: findings from a quasi-experimental cohort study. *J Epidemiol Commun H*. 2011;65(10):909-14. doi:10.1136/jech.2010.118133

5. Hansen M, Andersen TE, Armour C, Elklit A, Palic S, Mackrill T. PTSD-8: A Short PTSD Inventory. *Clin Pract Epidemiol Ment Health*. 2010;6:101-8. doi:10.2174/1745017901006010101

6. Hansen M, Andersen TE, Armour C, Elklit A, Palic S, Mackrill T. PTSD-8: A short PTSD inventory. United Kingdom: Bentham Science Publishers Ltd., 2010.

7. Andersen TE, Hansen M, Ravn SL, Seehuus R, Nielsen M, Vaegter HB. Validation of the PTSD-8 Scale in Chronic Pain Patients. *Pain Med*. 2018;19(7):1365-1372. doi:10.1093/pm/pnx166

8. Goodman R, Meltzer H, Bailey V. The Strengths and Difficulties Questionnaire: a pilot study on the validity of the self-report version. *Int Rev Psychiatr*. 2003;15(1-2):173-7. doi:10.1080/0954026021000046137

9. Goodman R, Meltzer H, Bailey V. The Strengths and Difficulties Questionnaire: a pilot study on the validity of the self-report version. *Eur Child Adoles Psy*. 1998;7(3):125-30.

10. Williamson A, Mcelduff P, Dadds M, et al. The Construct Validity of the Strengths and Difficulties Questionnaire for Aboriginal Children Living in Urban New South Wales, Australia. *Aust Psychol*. 2014;49(3):163-170. doi:10.1111/ap.12045

11. Coventry City Council. Strength and Difficulties Questionnaires (SDQ's) Practice Guidance, 2018.

© 2023 Guo L et al. *JAMA Network Open*.
