# Supplementary material for: Characterization of Premigration and Postmigration Multidomain Factors and Psychosocial Health Among Refugee Children and Adolescents After Resettlement in Australia
Source: JAMA Netw Open. 2023 Apr 6;6(4):e235841. doi: 10.1001/jamanetworkopen.2023.5841 (PMC10080375; doi:10.1001/jamanetworkopen.2023.5841)
Supplement: Supplement 2. — Data Sharing Statement [file jamanetwopen-e235841-s002.pdf]

Data Sharing Statement

Guo. Characterization of Premigration and Postmigration Multidomain Factors and Psychosocial Health Among Refugee Children and Adolescents After Resettlement in Australia. *JAMA Netw Open*. Published April 06, 2023.
doi:10.1001/jamanetworkopen.2023.5841

Data
Data available: Yes
Data types: Deidentified participant data
How to access data: All relevant data can be found in the following repository: https://bnla.aifs.gov.au/
When available: With publication

Supporting Documents
Document types: Statistical/analytic code
How to access documents: Statistical/analytic code can be provided upon the request to the corresponding author.
When available: With publication

Additional Information
Who can access the data: Researchers whose proposed use of the data has been approved
Types of analyses: Any purpose or for a specified purpose
Mechanisms of data availability: after approval of a proposal, or with a signed data access agreement
